# Effect of chemotherapeutic agents on natural transformation frequency in Acinetobacter baylyi

**DOI:** 10.1099/acmi.0.000733.v4

**Published:** 2024-07-10

**Authors:** Macaulay Winter, Michiel Vos, Angus Buckling, Pål Jarle Johnsen, Klaus Harms

**Affiliations:** 1 European Centre for Environment and Human Health, University of Exeter Medical School, Penryn Campus, Exeter TR10 9FE, UK; 2 Environment and Sustainability Institute, University of Exeter, Penryn Campus, Penryn TR10 9FE, UK; 3 Centre for Ecology & Conservation, University of Exeter, Penryn Campus, Exeter TR10 9FE, UK; 4 Microbial Pharmacology and Population Biology Research Group, Department of Pharmacy, Faculty of Health Sciences, UiT, The Arctic University of Norway, Tromsø, Norway

**Keywords:** *Acinetobacter baylyi*, antimicrobial resistance, cancer chemotherapeutic agents, natural transformation

## Abstract

Natural transformation is the ability of a bacterial cell to take up extracellular DNA which is subsequently available for recombination into the chromosome (or maintenance as an extrachromosomal element). Like other mechanisms of horizontal gene transfer, natural transformation is a significant driver for the dissemination of antimicrobial resistance. Recent studies have shown that many pharmaceutical compounds such as antidepressants and anti-inflammatory drugs can upregulate transformation frequency in the model species *Acinetobacter baylyi*. Chemotherapeutic compounds have been shown to increase the abundance of antimicrobial resistance genes and increase colonization rates of potentially pathogenic bacteria in patient gastrointestinal tracts, indicating an increased risk of infection and providing a pool of pathogenicity or resistance genes for transformable commensal bacteria. We here test for the effect of six cancer chemotherapeutic compounds on *A. baylyi* natural transformation frequency, finding two compounds, docetaxel and daunorubicin, to significantly decrease transformation frequency, and daunorubicin to also decrease growth rate significantly. Enhancing our understanding of the effect of chemotherapeutic compounds on the frequency of natural transformation could aid in preventing the horizontal spread of antimicrobial resistance genes.

## Data Summary

Supporting data and method of analysis with Supplementary Material for Effect of Chemotherapeutic Agents on Natural Transformation Frequency in *Acinetobacter baylyi* are deposited at 10.6084/m9.figshare.24468091 [1].

## Introduction

Antimicrobial resistance (AMR) is a global threat to modern medicine and is accelerated greatly by rapid dissemination of antimicrobial resistance genes via horizontal gene transfer (HGT) [2,6]. The increased prevalence of AMR has severe consequences for modern medicine; for instance, AMR infections were linked to an estimated 1.27 million deaths worldwide in 2019 [7]. Natural transformation, the process whereby prokaryotes take up extracellular DNA from the environment [8,10], is an underrecognized driver of AMR dissemination worldwide despite conferring the ability to acquire cell-free chromosomal DNA, plasmids and transposons [11]. While only approximately 80–90 species are known to be naturally transformable [10], the WHO’s list of priority multidrug-resistant pathogens is composed mostly of transformable species, indicating that this mechanism could be important in the acquisition of resistance [12].

Transformation frequency in bacteria can be up- or down-regulated in response to a range of stimuli [10,13]. A wide variety of anthropogenic pollutants including pharmaceutical products have been demonstrated to increase transformation frequencies, particularly in *Acinetobacter baylyi* [10,14,18]. For example, pharmaceutical compounds such as anti-inflammatory drugs [14] and antidepressants [19] can increase natural transformation frequency two- to threefold. However, no data are available on the possible effect of chemotherapy compounds on natural transformation. Chemotherapy compounds are cytotoxic agents which target a range of human cell functions which are often upregulated in – or unique to – malignant cells to induce cell death [20]. The use of chemotherapy compounds to treat malignancies in humans may lead to increased levels of AMR in gut microbiota through increasing rates of *de novo* mutation, HGT or by acting as a selective pressure [21,23]. Consequently, this may increase the risk of contracting resistant bloodstream infections, a cause of death in approximately 1 in 10 cancer patients [24,25]. A proposed mechanism for AMR acquisition in response to exposure to chemotherapy compounds is the induction of the SOS response pathway via genotoxic or cytotoxic damage which consequently leads to increased bacterial mutation rates [22,26, 27]. Although the SOS stress response pathway is atypical in *A. baylyi* [28,29], it is considered to be an inducer of competence for natural transformation [30], as it is in other species [31,33]. Therefore, exposure to chemotherapeutic compounds could be hypothesized to increase transformation frequencies.

Here, we use the model organism *A. baylyi* to test the effects of chemotherapeutic drugs on natural transformation. *A. baylyi* is a ubiquitous environmental bacterium capable of opportunistic infection [34,35], and is constitutively naturally transformable [13,36]. *Acinetobacter* species, particularly *A. baumannii*, can colonize human gastrointestinal systems [37] and can cause severe and even fatal infections in patients undergoing cancer chemotherapy [38,39]. We exposed *A. baylyi* to six chemotherapy compounds currently used to treat malignancies in humans to test for dose-dependent changes in transformation frequency and growth rate in *A. baylyi*. Each drug belongs to a different class with different mechanisms of action: cytarabine, a cytosine analogue [40]; daunorubicin, a DNA topoisomerase II inhibitor [41]; docetaxel, a disruptor of microtubule function [42]; exemestane, an aromatase inhibitor [43]; imatinib, a tyrosine kinase inhibitor [44]; and methotrexate, a folate synthesis inhibitor [45] (Table 1). As the varied pharmacokinetic properties of these six drugs can indicate differences in diffusion between blood plasma and tissues [46], a range of concentrations spanning previously measured blood plasma concentrations were used.

**Table 1.** List of the drugs used in this study, their mechanisms of action and concentrations reported for clinical samples

Drug	Mechanism of action	Clinically relevant concn
Cytarabine	Cytosine analogue	Blood plasma – 17.8 µg ml^−1^ [58]
Daunorubicin	Topoisomerase II inhibitor	Concentration inside leukaemic cells – 10.6 µg ml^−1^ [53]
Docetaxel	Disrupts microtubule function	Blood plasma – 2.42 ng ml^−1^ [51]
Exemestane	Aromatase inhibitor (oestrogen synthesis inhibitor)	Blood plasma – 4.1 ng ml^−1^ [59]
Imatinib	Tyrosine kinase inhibitor	Blood plasma – 1 µg ml^−1^ [60]
Methotrexate	Folate synthesis inhibitor	Blood plasma – 13.63 µg ml^−1^ [61]

## Methods

### Chemotherapeutic drugs

Cytarabine (Abcam), daunorubicin (Cayman Chemical Company), docetaxel (Cambridge Bioscience), exemestane (Merck), imatinib (Cambridge Bioscience) and methotrexate (Cayman Chemical Company) were stored at −20 °C in single-use aliquots dissolved in DMSO (Fisher) at 100× the concentration used in each treatment. Aliquots of drug stocks were added as 1 % of the final volume of culture to ensure an equal final concentration of DMSO across all treatments. DMSO at 1 % (v/v) had no effect on transformation frequency or growth rate in *A. baylyi*.

### Transformation assay

Genomic DNA as a substrate for natural transformation was isolated from an *A. baylyi* construct labelled with red fluorescence and spectinomycin resistance [47]. An isogenic transformable green fluorescent *apraR* wild-type *A. baylyi* ADP1 was grown overnight in LB broth (Formedium) and diluted fivefold into 2 ml of LB broth in a universal 30 ml container (see Winter *et al*. [47] for strain construction details). Cultures were amended with chemotherapeutic drugs on a log_10_ dilution range. In the no-drug control, DMSO was added to be consistent with the 1 % DMSO concentration in drug treatment groups. Spectinomycin resistance-conferring DNA was obtained by lysis following the Qiagen Genomic DNA Handbook (April 2012) protocol. DNA from the eluate was precipitated by adding two volumes of ice-cold isopropanol and centrifuged at 26 000 ***g*** for 15 min to pellet the DNA. DNA was dissolved in TE buffer to a final concentration of 342.4 ng µl^−1^ (Nanodrop 2000, Thermo Scientific) and frozen at −20 °C in single-use aliquots for addition to each experiment at a final concentration of 100 ng ml^−1^ for each sample. Samples were incubated at 30 °C and 180 r.p.m. for 5 h. Recipients and transformants were enumerated before and after incubation by plating on LB agar amended with 240 µg ml^−1^ apramycin (Duchefa), and LB agar amended with both 240 µg ml^−1^ apramycin and 360 µg ml^−1^ spectinomycin (Melford), respectively. Concentrations of antibiotics far exceeding the MICs required to inhibit *A. baylyi* growth were used to reduce the chance of false positives caused by contamination. All treatments were sampled at a minimum of sixfold biological replication. To determine the highest concentration of DMSO which had no observable effect on cell viability and growth, a transformation assay was conducted as above, but without chemotherapy compounds (five-fold dilution of *A. baylyi* into LB broth for 3 h at 30°C, 180 r.p.m., with 100 ng ml^−1^ DNA and 10, 1, 0.01, 0.001 or 0 % DMSO; data not shown).

### Statistical analyses

The effects of chemotherapy compound presence and concentration on *A. baylyi* transformation frequencies and growth rate were determined using Kruskal–Wallis and paired Wilcoxon testing, respectively. For analyses measuring growth rate, the Malthusian parameter was calculated and used as a response variable [48]. Fold changes in transformation frequency were calculated using the means of the respective compared treatment groups. In all analyses, *P* values of <0.05 were considered significant. False discovery rate adjustment for multiple testing was used in all instances where multiple tests were conducted in the same analysis.

## Results

### Contrasting effects of different chemotherapy drugs on natural transformation frequency in *A. baylyi*

**Fig. 1.**
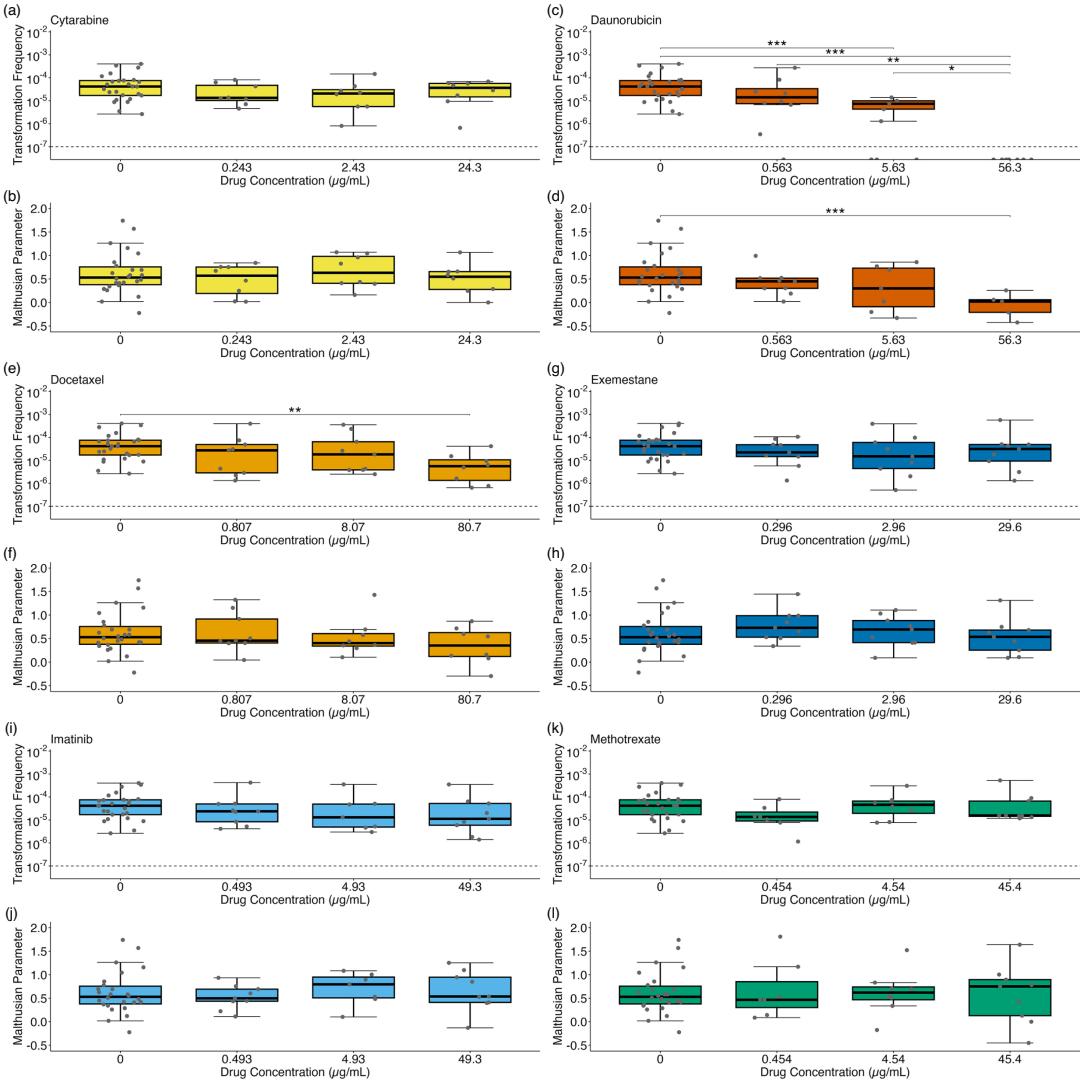
Effect of six chemotherapeutic agents on the transformation frequency and growth rate of *A. baylyi*. Effect of cytarabine on transformation frequency (**a**) and growth rate (**b**); daunorubicin on transformation frequency (**c**) and growth rate (**d**); docetaxel on transformation frequency (**e**) and growth rate (**f**); exemestane on transformation frequency (**g**) and growth rate (**h**); imatinib on transformation frequency (**i**) and growth rate (**j**); methotrexate on transformation frequency (**k**) and growth rate (**l**). Plotted points represent individual replicates and are horizontally scattered for improved visibility only. Values plotted below 10^−7^ (dashed line) indicate frequencies below the detectable limit (*P<0.05; **P<0.01; ***P<0.001; ****P<0.0001).

We tested the effect of six chemotherapeutic compounds on natural transformation and growth rate at varied concentrations pertaining to those found in patient blood plasma. Cytarabine was not observed to have a significant effect on transformation frequency (Kruskal–Wallis test, *H*=2.22, df=3, *P*=0.528; Fig. 1a) or growth rate (Kruskal–Wallis test, *H*=0.878, df=3, *P*=0.831; Fig. 1b). Daunorubicin resulted in significantly decreased transformation frequency (Kruskal–Wallis test, *H*=28.4, df=3, *P*<0.0001; Fig. 1c) and growth rate (Kruskal–Wallis test, *H*=17.3, df=3, *P*<0.001; Fig. 1d). Compared to the control group, transformation frequency of *A. baylyi* in 5.63 µg ml^−1^ daunorubicin significantly reduced by over 16-fold [Wilcoxon pairwise comparison, *H*=194, *P*<0.001; Fig. 1c and Table S1 (available in the online version of this article)]. Growth rate of *A. baylyi* in the presence of 56.3 µg ml^−1^ daunorubicin was significantly reduced compared to the control group (Wilcoxon pairwise comparison, *W*=200, *P*<0.001; Fig. 1d and Table S2), and is the only treatment group in this study where a net loss of cells was observed. Growth rates of *A. baylyi* in 0.563 and 5.63 µg ml^−1^ daunorubicin were not significantly different to the control group (Wilcoxon pairwise comparisons, *P*>0.05; Fig. 1d and Table S2). Transformation frequency of *A. baylyi* in 56.3 µg ml^−1^ daunorubicin was below the detectable limit (10^−7^) and significantly lower than all other concentrations and the no-drug control (Wilcoxon pairwise comparisons, *P*<0.05; Fig. 1c and Table S1). Docetaxel significantly decreased the transformation frequency over sevenfold at 80.7 µg ml^−1^ (Wilcoxon pairwise comparison, *W*=181, *P*<0.01; Fig. 1e and Table S3), but did not affect the growth rate (Kruskal–Wallis test, *H*=2.47, *P*=0.481, df=3; Fig. 1f). Exemestane did not have a significant effect on transformation frequency (Kruskal–Wallis test, *Η*=2.35, *P*=0.504, df=3; Fig. 1g) or growth rate (Kruskal–Wallis test, *Η*=2.56, *P*=0.465, df=3; Fig. 1h). Imatinib had no significant effect on transformation frequency (*Η*=3.51, *P*=0.319, df=3; Fig. 1i) or growth rate (Kruskal–Wallis test, *Η*=1.29, *P*=0.732, df=3; Fig. 1j). Methotrexate had no significant effect on transformation frequency (Kruskal–Wallis test, *Η*=3.71, *P*=0.294, df=3; Fig. 1k) or growth rate (Kruskal–Wallis test, *Η*=0.101, *P*=0.992, df=3; Fig. 1l).

## Discussion

In this study, six chemotherapeutic drugs with diverse mechanisms of action were tested for their effect on natural transformation and growth rate in *A. baylyi*. Four compounds, cytarabine, exemestane, imatinib and methotrexate, demonstrated no observable effect on natural transformation or growth at clinically relevant concentrations, while two compounds, docetaxel and daunorubicin, caused a dose-dependent decrease in transformation frequency, with daunorubicin significantly decreasing growth rate.

Docetaxel acts on eukaryotic cells by causing disruption of microtubule function leading to reduced cell proliferation [42]. If cell proliferation in *A. baylyi* was arrested by docetaxel, we expected to see a reduction in competence as competence is linked to growth phase in this species [36,49, 50]. Docetaxel negatively affected transformation frequency, but not growth rate, suggesting that its mechanism of action primarily affects transformation machinery and is not an indirect effect of reduced growth rate. Clinically relevant blood concentrations of docetaxel are around 2.42 ng ml^−1^ [51] and are therefore lower than those used in this study. Exposure to daunorubicin could in theory lead to altered rates of *gyrA* and *gyrB* transcription which are also upregulated in the SOS response in *A. baylyi* [19]. Daunorubicin showed strong effects on both growth rate and transformation frequency where transformation frequency reduced to below detectable levels at 56.3 µg ml^−1^ daunorubicin. As growth rate is unaffected at lower concentrations of daunorubicin, its effects on transformation may be independent of effects on growth rate, indicating that the mechanism of action of daunorubicin may act directly on DNA uptake machinery. Intracellular concentrations of daunorubicin may also be sufficient for the promotion of secondary structures of ssDNA internalized by cells caused by binding of the drug to ssDNA [52], thereby limiting the accessibility of ssDNA for recombination. The mean concentration of daunorubicin found in leukaemic cells during treatment is around 18.8 µmol l^−1^ [53] or 10.6 µg ml^−1^ which is within the range tested here and so the observed effects may be clinically relevant. However, it is unclear how concentrations of cancer chemotherapeutics in blood are related to those in blood serum. To our knowledge, only one study to date has attempted to estimate non-antibiotic drug concentrations in the gut [54], but exclusively considered oral drug administration. As both daunorubicin and docetaxel are administered intravenously, we cannot estimate the concentrations at which these drugs are found in the gut and thus the clinical relevance of the concentrations tested in this study. *A. baylyi* is a ubiquitous environmental bacterium which may come into contact with cancer chemotherapeutic pollutants in wastewater systems [28,55].

The routine use of last-resort antibiotics as a prophylactic treatment for cancer patients increases the resistance of gut microbiota to those antibiotics [56] and has been known to increase colonization of potentially pathogenic bacteria in the gut [57]. This can lead to infection but could also potentially act as a donor pool of resistance or pathogenicity genes to resident species that are able to engage in natural transformation. The limited effect the drugs tested in this study have on transformation frequency at putatively clinically relevant concentrations is favourable, as it may reduce or not affect the rate of transformation events which can lead to the acquisition of traits which are beneficial to pathogens. Future work is needed to close the knowledge gap on *in vitro* concentrations of non-antibiotic drugs in different organ systems and different human-associated bacteria. Additionally, our data identify a need to further investigate the effects and mechanisms of both these and currently untested chemotherapeutic drugs to help monitor and establish preventative measures which can limit the spread of AMR genes.

## supplementary material

10.1099/acmi.0.000733.v4 Uncited Table S1.

## Abbreviations

AMR: antimicrobial resistance
DMSO: dimethylsulfoxide
HGT: horizontal gene transfer
ssDNA: single stranded DNA
WHO: World Health Organisation
