# Supplementary material for: Effect of chemotherapeutic agents on natural transformation frequency in Acinetobacter baylyi
Source: Access Microbiol. 2024 Jul 10;6(7):000733.v4. doi: 10.1099/acmi.0.000733.v4 (PMC11318045; doi:10.1099/acmi.0.000733.v4)
Supplement: Uncited Table S1. [file acmi-6-00733-s001.pdf]

Effect of Chemotherapeutic Agents on Natural Transformation Frequency in *Acinetobacter baylyi*

Supplemental Material

Supplemental table 1. Wilcoxon paired comparisons of *A. baylyi* transformation frequency in varying concentrations of daunorubicin. A 0.95 confidence interval was used for all samples.

Concentration 1 (µg/mL)	Concentration 2 (µg/mL)	n1	n2	W statistic	p value	Adjusted p value	Significance
0	0.563	26	9	162	0.093	0.1	ns
0	5.63	26	8	194	0.000278	0.000834	***
0	56.3	26	8	208	0.0000234	0.00014	***
0.563	5.63	9	8	53.5	0.1	0.1	ns
0.563	56.3	9	8	68	0.001	0.002	**
5.63	56.3	8	8	52	0.013	0.0195	*

Supplemental table 2. Wilcoxon paired comparisons of *A. baylyi* growth rate in varying concentrations of daunorubicin. A 0.95 confidence interval was used for all samples.

Concentration 1 (µg/mL)	Concentration 2 (µg/mL)	n1	n2	W statistic	p value	Adjusted p value	Significance
0	0.563	26	9	151	0.206	0.247	ns
0	5.63	26	8	142	0.128	0.195	ns
0	56.3	26	8	200	0.000105	0.00063	***
0.563	5.63	9	8	45	0.423	0.423	ns
0.563	56.3	9	8	69	0.000576	0.00173	**
5.63	56.3	8	8	47	0.13	0.195	ns

Supplemental table 3. Wilcoxon paired comparisons of *A. baylyi* transformation frequency in varying concentrations of docetaxel. A 0.95 confidence interval was used for all samples.

Concentration 1 (µg/mL)	Concentration 2 (µg/mL)	n1	n2	W statistic	p value	Adjusted p value	Significance
0	0.807	26	9	151	0.21	0.354	ns
0	8.07	26	9	145	0.305	0.366	ns
0	80.7	26	8	181	0.000976	0.00586	**
0.807	8.07	9	9	39	0.931	0.931	ns
0.807	80.7	9	8	49	0.236	0.354	ns
8.07	80.7	9	8	50	0.2	0.354	ns
